# Risk stratification of post-MI patients for ICD implantation using texture analysis to quantify heterogeneity of scar

**DOI:** 10.1186/1532-429X-17-S1-Q14

**Published:** 2015-02-03

**Authors:** Noor Ali, Gregory Mullen, Amedeo Chiribiri

**Affiliations:** 1 Imaging Sciences, King's College London, London, UK; 2 Cardiovascular (imaging) department, King's College London, London, UK

## Background

Following myocardial infarction (MI), patients are at risk of sudden cardiac death (SCD) due to ventricular arrhythmia, which can be prevented with an implantable cardioverter-defibrillator (ICD). Recommendations for ICD implantation is currently based on left ventricular ejection fraction (LVEF), however less than a quarter of patients who receive ICDs based on LVEF have appropriate therapy. Heterogeneity of scar has been implicated in the development of re-entrant arrhythmias and SCD. Texture analysis (TA) is a method of quantifying heterogeneity of tissues in imaging, usually using statistical-based methods to evaluate distribution of grey-level pixels. This study aimed to determine whether TA could be used to quantify heterogeneity of scar as a method of accurately risk stratifying patients.

## Methods

This was a retrospective blinded analysis of late-gadolinium enhanced cardiac magnetic resonance (LGE-CMR) images. Twenty post-MI patients who received ICDs were followed up for up to 686 days after implantation. Ten of these patients went on to have events and were categorised as high risk, while the remaining ten had no events on follow up and were categorised as low risk. TA was performed on regions of interest delineating scar on short axis LGE images of all patients, which involved an initial filtration step, highlighting objects of 2-6mm radii using a laplacian of Gaussian filter, denoted by the term spatial scaled factor (SSF) 2-6, followed by histogram analysis of pixel intensity from which a set of statistical parameters could be derived. Further statistical analysis was performed to determine group-wise differences in TA statistical parameters as well as survival analysis to predict time to event.

## Results

Significantly different (p<0.05) statistical parameters in group-wise comparison included higher kurtosis of the histogram in the high risk group when SSF 5 and 6 were applied, and more negative skewness of the histogram in the high risk group when no filter was applied. Receiver operating characteristic (ROC) curves were drawn for these three parameters and coordinates with the highest combined sensitivity and specificity were extracted for use as threshold values in Kaplan-Meier graphs to compare time to event, which were statistically different in all three cases (p<0.05).

## Conclusions

This preliminary study showed that TA of scar identified by LGE in CMR images revealed significant differences in specific statistical parameters between high and low risk groups, as well as significant differences in time to event. There is therefore basis for expanding the analysis and comparing with other clinical and CMR parameters to explore potential for a more accurate method of risk stratifying post-MI patients for ICD implantation.

## Funding

N/A.

**Figure 1:**
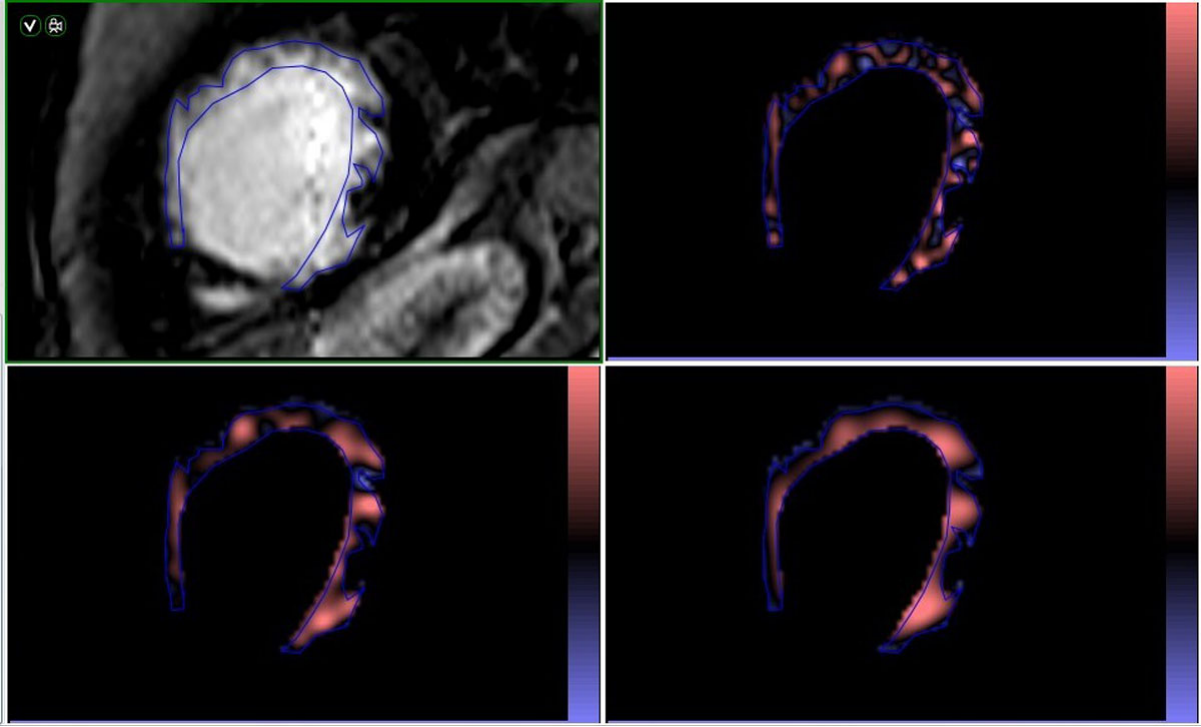
Short axis LGE-CMR image slice with ROI drawn around scar (top left) with highlighted features of spatial scaled factor 2 (top right), 4 (bottom left) and 6 (bottom right).

**Table 1:** Significantly different texture analysis statistical parameters indicating increased scar heterogeneity in high risk patients

	Mean	SD	Entropy	MPP	Skewness	Kurtosis
SSF 0	0.481	0.912	0.912	0.971	0.035*	0.315

SSF 2	0.481	0.971	0.853	0.853	0.796	0.739

SSF 3	0.353	0.971	0.912	0.853	0.353	0.684

SSF 4	0.315	0.971	0.971	0.853	0.123	0.089

SSF 5	0.218	0.853	0.853	0.796	0.075	0.011*

SSF 6	0.28	0.971	0.796	0.796	0.075	0.023*

2-tailed significance of Mann Whitney tests comparing TA statistical parameters of high and low risk patients when different spatial scaled factors (SSF) applied. *Significant parameters (p<0.05)

